# Health of the City

**Published:** 1854-06

**Authors:** 


### HEALTH OF THE CITY.

Louisville continues healthy, for the season, which has been wet, and is now excessively hot. If any cases of cholera have originated in the city, they have thus far been few and scattering. Dysentery prevails to some extent, with diarrhea, cholera morbus, and cholera infantum. There is plainly a tendency to bowel complaints, as might be expected in such weather. Cholera is reported to have appeared in many places, in this State and in Tennessee, but we do not hear that it is anywhere committing such ravages as attended the march of the disease in former years.

